# Potential Health Benefits of a Diet Rich in Organic Fruit and Vegetables versus a Diet Based on Conventional Produce: A Systematic Review

**DOI:** 10.1093/nutrit/nuae104

**Published:** 2024-08-05

**Authors:** Nathalie Komati, Jean-Pierre Cravedi, Jean-Michel Lecerf, Luc P Belzunces, Delphine Tailliez, Claire Chambrier, Johanna Calvarin, Marie-Josèphe Amiot

**Affiliations:** The Agency for Research and Information on Fruit and Vegetables (APRIFEL), Paris 75017, France; The Agency for Research and Information on Fruit and Vegetables (APRIFEL), Paris 75017, France; Nutrition & Physical Activity Department, Institut Pasteur de Lille, Lille 59000, France; INRAE, Laboratoire de Toxicologie Environnementale, UR 406 A&E, Avignon Cedex 9 84914, France; The Agency for Research and Information on Fruit and Vegetables (APRIFEL), Paris 75017, France; The Agency for Research and Information on Fruit and Vegetables (APRIFEL), Paris 75017, France; The Agency for Research and Information on Fruit and Vegetables (APRIFEL), Paris 75017, France; MoISA, Université de Montpellier, CIHEAM-IAMM, CIRAD, INRAE, Institut Agro, IRD, Montpellier 34000, France

**Keywords:** diet, fruit and vegetables, organic, conventional, health outcome

## Abstract

**Context:**

Over the past decade, the production and consumption of organic food (OF) have received increasing interest. Scientific studies have shown better quality of organic fruit and vegetables (FV) in terms of nutrients and pesticide contents, but it appears difficult to conclude if there are potentially greater health benefits of these products compared with conventional food (CF).

**Objective:**

To determine whether the current scientific literature demonstrates that a diet rich in organic FV is healthier than 1 based on conventional produce.

**Methods:**

A systematic search was conducted using the PubMed and Web of Science databases for articles published between January 2003 and December 2022. Articles were analyzed uniformly by 2 reviewer, using a specific template summary sheet, and scored from 1 to 5. The level of evidence and the quality of studies in humans were assessed using the Jadad score and the French National Authority for Health method.

**Results:**

A total of 12 human studies were included. Studies often reported contradictory or even opposite results, with methodological limitations. Only 6 of the 12 studies found significant associations between OF and the health outcomes evaluated.

**Conclusion:**

The current data do not enable a firm conclusion about a greater health benefit for a diet rich in FV based on products grown organically compared with conventional farming. There is a paucity of available data and considerable heterogeneity in study designs (participants, exposures, durations, health outcomes, and residual confounding factors). Well-designed interventional studies are required.

## INTRODUCTION

Over the past decade, there has been increasing interest in the production and consumption of organic food (OF), even though a decline caused by high inflation combined with increasing food costs has been observed in the past 2 years.^1^ European consumers, nevertheless, are now spending more on OF than in the past. Per capita, consumer spending on OF has doubled in the past decade (€35.5/person in 2009 vs €84.2/person in 2019).^2^ Baby foods are the most frequently consumed OFs in Europe, followed by eggs, fruit and vegetables (FV), and dairy products. In the United States, FV were reported to be in the top position for OF sales.^2^ Some countries have recommended OF products in their dietary guidelines for human health benefits (Brazil and France) and also for sustainability (Sweden and Slovakia).^3–5^ Major motivators to purchase OF products are food safety, human health, animal welfare, environmental concerns, and higher nutrient content, alongside certain sensory attributes, including taste, freshness and appearance. Organic FV are perceived as more sustainable and have more nutrient contents and zero or lower conventional pesticide residues compared with conventional food (CF).^6^ However, the higher price of OF products remains the main barrier to their purchase. Other barriers are related to limited availability on the market, current satisfaction with conventional food along with the perception that the benefits of OF might not be higher, lack of trust in organic labels due to their number and complexity, lack of promotion, and the general misunderstanding of organic production processes.^7–9^

A recent review showed that organic FV are characterized by a slightly higher content in polyphenols and vitamin C, certain minerals (iron, magnesium) and lower levels of pesticide residues, but, in some cases, might contain higher levels of mycotoxins and allergens (eg, profilin and Bet v 1).^10^ Even though scientific studies have shown better quality for organic FV in terms of nutrient and pesticide contents, it appears difficult to conclude if the health benefits of these products are greater. To our knowledge, 3 systematic reviews were conducted in 2010, 2012, and 2019 to assess the effect of OF consumption on health outcomes.^11–13^ None of these studies showed a real benefit, due to heterogeneity and bias. The literature has expanded during the past decade, however, so the present review was conducted to determine whether the current scientific literature demonstrates that a diet rich in organic FV is healthier than a diet containing conventional produce.

## METHODS

A Preferred Reporting Items for Systematic Reviews and Meta-Analysis (PRISMA) approach was adopted (see checklist in Table S1).^14^ The systematic review protocol was not registered.

### Literature search

A systematic review of the literature was conducted using the PubMed and Web of Science databases to find articles published between January 2003 and December 2022. Relevant keywords included terms related to dietary intake from organic and/or conventional production, in combination with terms related to health outcomes (ie, chronic disease, human health, obesity, diabetes, and cancer). Search terms were adjusted slightly for each database and the filter “TS” for “Topic” was applied (ie, term included in the title of the article and/or abstract and/or keywords). The following terms were excluded: “consumer,” “behaviour,” “perception,” “cells,” and “organoleptic” (Table S2).

### Selection criteria and data extraction

Inclusion and exclusion criteria are listed in Table 1 based on the population, intervention, comparison, outcome (PICO) approach. A summary sheet was created to uniformly analyze the articles. Seven reviewers with different and complementary expertise were assigned to analyze the articles. Each full article was reviewed independently by 2 reviewers for inclusion in the study, based on its relevance and eligibility criteria. A score from 1 to 5 (5 being the highest in terms of relevance and eligibility criteria) was given by both reviewers to each article. Based on the difference between the 2 reviewers’ scores (scores 1 and 2), specific rules were applied to decide whether the article would be retained, rejected, or discussed by the 2 reviewers. Any disagreement between the reviewers on whether an article should be retained or rejected was discussed in a meeting with the 7 experts on the review committee.

**Table 1.** PICOS Criteria for Inclusion of Studies

	Inclusion criteria	Exclusion criteria
Language	English	Other than English
Population	Human Any age	In vitro study Animal study population
Intervention	Randomized controlled trial Noncontrolled trial Prospective or retrospective cohort study Case-control study Cross-sectional study	In vitro study Study in animals
Comparison	Effects of organic food on health Effects of conventional food on health Comparison of organic food vs conventional food on health	None
Outcomes	Direct effects on human health (eg, on chronic diseases, immunity)	Article assessing contents in nutrients and/or pesticide residues and/or based on health risk assessment Article assessing nutritional biomarkers without evaluating a health impact Article related to plant health and metabolism Article based on consumer perception

### Scoring human studies

The level of evidence and the quality of studies were assessed using the Jadad score (0-5 scale), a widely used scale, named after the physician Alex Jadad, to assess the methodological quality of a clinical trial for the degree of reliability of studies^15^ and the French National Authority for Health method (A-C scale) for the level of evidence in the literature (Table S3).^16^

## RESULTS

### Overview of studies

**Figure 1.**
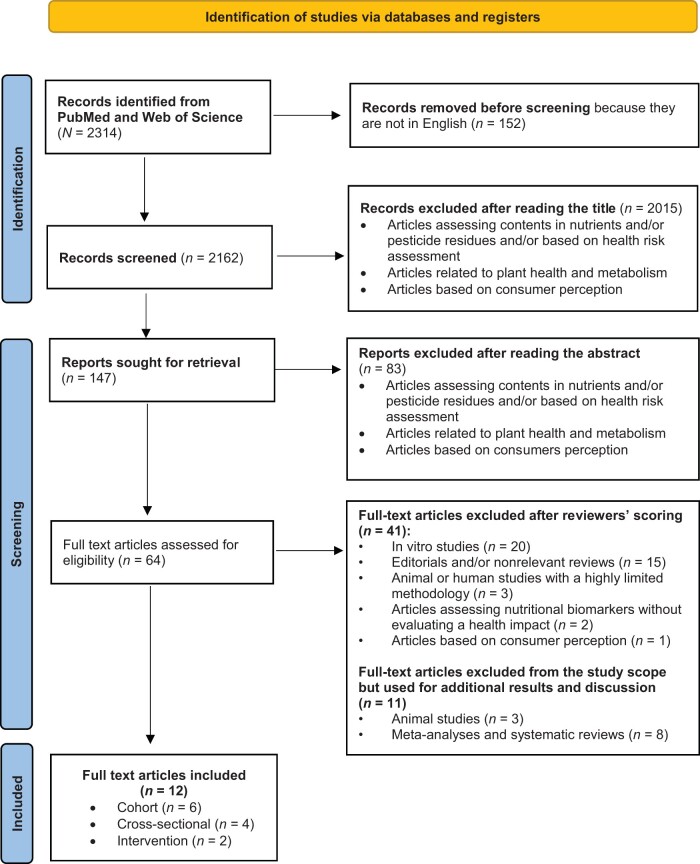
Preferred Reporting Items for Systematic Reviews and Meta-Analysis (PRISMA) Flow Diagram of Search Procedure

The original systematic search strategy included a step to identify relevant studies. First, 2314 articles were identified, of which 64 were retained as potentially relevant after reading the abstract. An independent examination of the full text of these 64 articles by 2 reviewers resulted in the exclusion of 41 articles. One of the main reasons for exclusion was that these articles presented no study outcome with a direct relevance for health and rather corresponded to a comparison or an assessment of nutrient contents. Eleven articles (*n* = 8 meta-analyses and 3 animal studies) were excluded from the review but were analyzed to support human studies in the ”Discussion.” A final total of 12 human studies were included. A flowchart of the article selection process is presented in Figure 1.^14^

### Studies in humans

The 12 human in vivo studies included 6 prospective, 4 cross-sectional, and 2 interventional studies. They were described using the following parameters: (1) author and year of publication; (2) study design, including country of origin and duration; (3) target population (sample size, mean age, percentage of the cohort that was female); (4) exposure; (5) outcomes; (6) results and statistical difference; and (7) conclusions (Table 2).

**Table 2.** Human Studies (*n* = 12) Included in the Systematic Review

Reference	Study type; name; country; follow up	No. of participants; mean age (SD) y; % female	Exposure	Outcomes	Results and statistical significance	Conclusions	Jadad score	HAS score
Baudry, 2018^17^	Prospective; NutriNet-Santé; France; 4.56 (SD 2.08) y	68 946; 44.2 y; 78.0% female	Consumption frequency of labelled OF for 16 products: never, occasionally, or most of the time OFS: 0-32 points Quartiles according to OFS: Q1 = 0.72 (0.82); Q2 = 4.95 (1.41); Q3 = 10.36 (1.69); Q4 = 19.36 (4.28)	Overall and specific cancers	Significant reduction of overall cancer risk Q4 vs Q1 (HR, 0.75; 95% CI, 0.63-0.88; *P* = .001 for trend; HR for a 5-point increase, 0.92; 95% CI, 0.88-0.96; *P *<* *.001) Lower risk (95% CI) of postmenopausal breast cancer 0.91 (0.83-1.01; *P *=* *.07), non-Hodgkin's lymphoma 0.75 (0.60-0.93; *P *=* *.009), and lymphoma 0.75 (0.60-0.93; *P *=* *.03) The relationship with breast cancer disappeared when the analysis was carried out with plant-based foods likely to contain pesticide residues.	A higher frequency of OF consumption was associated with a reduced risk of cancer. Residual confounding from unmeasured factors or inaccuracy in the assessment of some covariates cannot be totally excluded.	3	C
Kesse-Guyot, 2020^18^	Prospective; NutriNet-Santé; France; 4 y	33 256; 53 y (14); 76% female	Participants completed a 198-item FFQ and an FFQ on 7 OFs; 3 groups of OF purchase (<50%; 50%-90%; >90% organic). The proportion of OF in the diet was computed in 5% increments and as sex-specific quintiles (the lowest quintile as reference).	T2D	An increase of 5 points in the proportion of OF in the diet was associated with a 3% decrease in T2D risk. Reduction of 35% of T2D Q5 vs Q1 (95% CI, 0.43-0.97).	Higher OF consumption was associated with lower risk of T2D.	0	C
Simões-Wüst, 2017^19^	Prospective KOALA Birth Cohort Study; the Netherlands; NA	1339 Pregnant women; 32.0 y (3.6)	OF consumption was based on purchase, classifying 3 groups: <50%, 50%-90%, >90%, and compared with the conventional group as reference.	Maternal prepregnancy BMI, hypertension, diabetes in pregnancy, and several blood biomarkers: ferritin, homocysteine, LDL-cholesterol, fatty acids 25(OH)D	Average prepregnancy BMI was significantly lower in OF groups than in conventional group: <50% OF group (mean BMI 22.9 [SD 3.36] vs 23.8 [SD 3.92] kg/m^2^ in the conventional group; *P *<* *.001). Biomarkers: OF had a markedly lower EA: VA (18:1*n*-9, *trans*: 18:1*n*-7, *trans*) a marker of industrial vs natural origin of dietary trans-fatty acid, but lost significance when healthy diet indicators were added to the adjusted model. Blood 25(OH)D levels were lower in both OF groups >50% and remained significant even after adjustment (95% CI, –8.457 to 0.818 [group 50%-90% organic]; 95% CI, –10.681 to 2.185 [group >90%]; *P *<* *.01).	Consumption of OF during pregnancy was associated with several health-related characteristics and blood biomarkers, explained in part by food patterns accompanying OF consumption.	0	C
Bradbury, 2014^21^	Prospective; The Million Women Study; United Kingdom; 12 y	623 080; 59.4 y (4.9); 100% female	Questionnaire to assess frequency of OF consumption: never, sometimes, usually and always	All cancers combined (except nonmelanoma skin cancer) and 16 most common types of cancer in women	No association with a reduction in the incidence of all cancer (n=53 769 cases in total) (for usually/always vs never: RR, 1.03; 95% CI, 0.99-1.07), soft tissue sarcoma (RR, 1.37; 95% CI, 0.82-2.27), or breast cancer (RR, 1.09; 95% CI, 1.02-1.15), but inverse association with non-Hodgkin’s lymphoma incidence (RR, 0.79; 95% CI, 0.65-0.96)	No reduced risk of cancer overall or for 16 specific cancer sites or types among women who usually or always consume OF. But reduced risk of non-Hodgkin’s lymphoma	0	C
Torjusen, 2014^20^	Prospective; Norwegian Mother and Child Cohort Study; Norway; 6 y	28 192; 28.1 y (4.6); pregnant women	FFQ with 6 questions to assess OF consumption Scoring from 0 to 3 corresponding to “never or seldom,” “sometimes,” “often,” or “mostly,” respectively	Preeclampsia risk	Lower risk of preeclampsia in women who reported eating organically grown vegetables often or mostly (adjusted OR, 0.79; 95% CI, 0.62-0.99; *P *=* *.043) No associations were found for high intake of organic fruit, cereals, eggs or milk, or a combined index reflecting organic consumption.	Only consumption of organic vegetables during pregnancy was associated with a reduced risk of preeclampsia.	0	C
Kummeling, 2008^22^	Prospective KOALA Birth Cohort Study; the Netherlands; 2 y	2764 infants; 49% female	OF consumption was reported by parents: diet was defined as conventional (<50% organic), moderately organic (50%-90% organic), and strictly organic (>90% organic)	Atopic manifestations in the first 2 y of life	Lower eczema risk associated with consumption of organic dairy products (OR, 0.64; 95% CI , 0.44-0.93) No association between organic meat, FV, or eggs, or the proportion of organic products within the total diet and the development of eczema, wheeze, or atopic sensitization	Consumption of organic dairy products, within the context of an OD, is associated with a lower risk of eczema development.	1	C
Ludwig-Borycz, 2021^23^	Cross-sectional; United States; 3 y	3815 (including 50 from the Health and Retirement Study); 64.3 y; 54.4% female	Dietary data consumption with FFQ, and a specific question on OF consumed in the past year (yes/no): milk, eggs, meat, fruit (fresh or frozen), vegetables (fresh or frozen), bread or cereals, frozen prepared meals, or other. Using A-MedDiet for adjustment	Biomarkers of inflammation: CRP and CysC	CRP was inversely associated with consuming OF after adjustment of confounding factors. The association was attenuated with the addition of vegetarian status (OR, 0.799; 95% CI, 0.652-0.979) and the A-MedDiet (OR, 0.814; 95% CI, 0.673-0.984). CysC lost statistical significance after adjustment (OR, 0.982; 95% CI, 0.764-1.262). Association between OF consumption and log [CRP] was driven primarily by milk, FV, and cereals.	OF consumption is inversely associated with biomarkers of inflammation CRP and CysC, although residual confounding by healthy eating and socioeconomic status cannot be excluded.	0	C
Sun, 2018^26^	Cross-sectional; National Health and Nutrition Examination Survey; USA; NA	8199; 49.7 y (0.36)	Purchase of OF (yes/no) and the frequency of OF purchase: most of the time, sometimes, or rarely.	T2D (self-reported physician diagnosis or a hemoglobin A_1c_ level ≥6.5%, or both)	OR of T2D associated with OF purchase in the past 30 d was 0.80 (95% CI, 0.69-0.94; *P *=* *.01) after adjustment. Associations were significant for organic milk, eggs, and meats, but not for organic FV.	Frequent purchase of OF, particularly organic milk, eggs, and meats, was inversely associated with diabetes in US adults.	0	C
Baudry, 2018^24^	Cross-sectional; NutriNet-Santé study; France; NA	8174; 58.16 y (12.34)	An OF FFQ to assess the frequency of OF consumption: never, rarely, half of the time, often, and always (translated into quantitative data: to weight 0, 0.25, 0.5, 0.75, and 1, respectively) Assessment of OF proportion in 16 main food groups Population divided in tertiles according to proportion of OF in the whole diet (ratio): tertile (T) 1: 0.04; T2: 0.24; T3: 0.62	MetS prevalence	Higher OF consumption was negatively associated with MetS prevalence: T3 vs T1 (adjusted prevalence ratio, 0.69; 95% CI, 0.61-0.78; *P*<.0001). The negative association was observed, particularly when the proportion of plant-based OF increased (including FV, starchy foods, whole-grain products, and oil; *P*≤.0005 for all for linear contrast).	Higher OF consumption was associated with a lower probability of having MetS.	0	C
Gosling, 2021^25^	Cross-sectional; INCA3; France; NA	1775 children and adolescents (4-17 y); 2121 adults (18-79 y)	OF FFQ during the past 12 mo (always, often, rarely, never, did not eat, or do not know) for 12 food groups Consumption was scored “always” = 3 points, “often” = 2, “rarely” = 1, “never”, “did not eat” = 0, and as a missing value otherwise.	BMI and obesity	OF consumption was significantly negatively correlated with BMI and obesity (*P* <0.01 for all), even after adjustment.	Inverse association of OF consumption with both BMI and obesity during childhood and adulthood	0	C
Grinder-Pedersen, 2003^27^	Double-blinded, randomized, and crossover; Denmark; 22 d	16; 35 y; 62.5% female	Period 1: OD; period 2: CD; separated by a 3-wk wash-out period with a standard diet The menus and quantities of food used in the 2 diets were identical.	Urinary excretion of 5 flavonoids Markers of antioxidative defense: superoxide dismutase, glutathione peroxidase, glutathione reductase, and catalase in erythrocytes, Trolox equivalent antioxidant capacity, and the ferric reducing ability of plasma malondialdehyde	Urinary excretion (μg/24 h) of quercetin and kaempferol was significantly higher after intake of the OD compared with the CD (*P *<* *.05); no differences were seen between the 2 intervention periods with respect to the other measured flavonoids. The proportions of the excreted flavonoids were similar in both interventions. Most markers of antioxidative defense did not differ between both diets.	Growing conditions of FV impact the content of flavonoids, which results in higher urinary excretion of the major dietary flavonoids with an OD. Observed effects may originate from the varietal differences between the organic and conventional produce rather than from the differences in handling procedures, including pesticide use.	4	B
Hurtado-Barroso, 2019^28^	Randomized, controlled and cross-over; Spain; 4 mo	19 healthy participants; 30 y; 53% females	OD intervention (>80% organic products) followed by a CD (no OF allowed), each for 4 wk, separated by a 2-mo washout period	Biological status: minerals and heavy metals, bioactive compounds including phenolic acids (4-HBA), and carotenes	Significant higher content (3 times) of 4-HBA in the urine of those consuming OD (*P *=* *.028) No significant differences for other phenols No changes in carotenoids and inorganic elements in blood	A significant difference was only noted in the concentration of 4-HBA after OD. No changes were observed in the rest of the bioactive compounds nor in the other health-related biomarkers, or in the results of minerals and heavy metals. The relationship between OF or CF and the concentration of bioactives should be further researched.	3	B

*Abbreviations:* A-MedDiet, alternative Mediterranean diet score; BMI, body mass index; CD, conventional diet; CRP, C-reactive protein; CysC, cystatin C; EA, elaidic acid; FFQ, food frequency questionnaire; FV, fruits and vegetables; HAS, French National Authority for Health; HR, hazard ratio; LDL, low-density lipoprotein; MetS, metabolic syndrome; OD, organic diet; OF, organic food; OFS, organic foods score; OR, odds ratio; RR, relative risk; T2D, type 2 diabetes; VA, vaccenic acid; 4-HBA, 4-hydroxybenzoic acid.

#### Exposure and target population

All 6 prospective studies reported the effects of OF consumption as part of the whole diet using a food frequency questionnaire with specific questions related to OF. The proportion of OF in the diet was then computed as a quartile or quintile, or with a specific score, depending on the study (Table 2, “Exposure” column). Two prospective studies were conducted with adults recruited from the general population,^17^^,^^18^ 2 others focused on pregnant women,^19^^,^^20^ 1 was conducted with nonpregnant women,^21^ and 1 with infants.^22^ For the 4 cross-sectional studies, dietary intake and OF consumption were assessed in 3.^23–25^ One study evaluated the frequency of OF purchase instead of consumption.^26^ Adults were the target population in 3 studies; the fourth study^25^ also included children and adolescents. Both interventional studies (randomized, controlled, and crossover) were conducted with adults. They consisted of administering an organic diet (OD) during 2 periods, separated by a washout period in which a conventional diet (CD) was provided. In the first interventional study, the intervention periods lasted 22 days and the washout period 3 weeks.^27^ In the second study, the intervention periods lasted 28 days and the washout period 2 months.^28^

#### Health outcomes

Six of the 12 human studies found significant associations between OF consumption and the health outcome evaluated (Table 2). Two prospective studies had “overall and specific cancer” as an outcome. The One Million study^21^ is the pioneer study, with a large sample size and long follow-up, but data on exposure to pesticides remain imprecise. Regarding the increased risk of breast cancer in women consuming OF, the findings from the One Million study contrast with those of the NutriNet-Santé study, in which the sample size was 10 times smaller and the duration of exposure 2 times shorter. Importantly, in the NutriNet-Santé study,^17^ those who consumed OF the most had a lower overall risk of cancer than nonconsumers of OF. In absolute terms, the study’s findings were that 2.26% of non-OF consumers developed cancer compared with 1.6% of OF consumers. When analyzed by type of cancer, an association was found only for postmenopausal breast cancer, non-Hodgkin’s lymphoma, and all lymphomas. When a simplified food score was applied to plant-based foods, which are the only foods likely to contain pesticide residues (ie, fruit, vegetables, soya products, bread, cereals, and flour), the relationship with postmenopausal breast cancer disappeared in women.

One prospective and 1 cross-sectional study examined type 2 diabetes.^18^^,^^26^ Kesse-Guyot et al^18^ found that OF consumers had a reduced risk, but the authors concluded the observed effect was mainly due to the lifestyle profile of OF consumers and more generally, of the NutriNet-Santé cohort participants, known to have a healthy lifestyle and a balanced diet.^18^

Health outcomes related to pregnancy were studied in two prospective studies. Simões-Wüst et al^19^ evaluated the effect of OF consumption on maternal prepregnancy, body mass index (BMI), hypertension, and diabetes in pregnancy, and several blood biomarkers of pregnant women; Torjusen et al^20^ assessed the effect on the risk of preeclampsia during pregnancy.^20^ The latter study was inconclusive regarding the role of OF in preeclampsia prevention: no association was found between OF, as a whole dietary pattern, and the risk of preeclampsia, but an inverse association was only found between the consumption of organic vegetables and the risk of preeclampsia. This may be explained by the presence of specific nutrients in vegetables known for their benefits in preventing preeclampsia.^29^

A prospective study evaluated the association between early-life OF consumption and the development of atopic outcomes (namely, eczema and wheeze) in the first 2 years of life.^22^ According to this work, OF consumption during pregnancy has no effect on the risk of subsequent allergy (before 2 years of age); only the consumption of organic milk seems to have a beneficial effect on eczema. One cross-sectional study evaluated the effect of OF consumption on inflammation biomarkers (C-reactive protein and cystatin-C).^23^ Another study evaluated the effect of OF on BMI and obesity,^25^ and the third study looked at the prevalence of metabolic syndrome (MetS).^24^ Based on the results of the only study evaluating the MetS risk,^24^ a possible inverse relationship between OF and MetS can be concluded, but the dietary data were not specified in that study (eg, FV consumption). Although there is an adjustment on the food quality score, the role of lifestyle cannot be excluded, nor the contribution of pesticides, but the mechanistic hypotheses are weak, and the exposure remains approximate. Interventional studies, therefore, would be valuable.

The first interventional study assessed the effect of OF consumption on excretion of flavonoids and on markers of antioxidative defense in humans,^27^ and the second interventional study focused on biological parameters, inorganic elements, bioactive compounds, and phenolic acids and carotenes.^28^

#### Level of evidence

The Jadad score showed that interventional studies^27^^,^^28^ are the most reliable (score 4), whereas prospective and cross-sectional studies, except 1 with a score 3,^17^ are the least reliable (score 0 or 1). According to the French National Authority for Health method, interventional studies were also those with the highest level of evidence (score B) (Table 1 and Table S3).

## DISCUSSION

Based on the available data, the present review does not enable a firm conclusion to be drawn about the health benefit of a diet rich in FV based on products grown organically compared with conventionally. Among the 12 studies selected, 6 did not find any significant association between OF consumption and the health outcome(s) considered, and 6 found significant associations. A significant reduction of overall cancer risk for high consumers of OF was reported by Baudry et al.^17^ Simões-Wüst et al^19^ found that average prepregnancy BMI was significantly lower in the OF group than in the CF group. A significant inverse association was reported between OF consumption and the log of C-reactive protein concentration and log of cystatin-C concentration.,^23^ MetS prevalence,^24^ and BMI and obesity during childhood and adulthood.^25^ Finally, Sun et al^26^ reported that participants who purchased OF were significantly less likely to have diabetes, with more pronounced associations found for organic milk, eggs, and meats than for organic FV.

The studies in humans included in this review have limitations. For instance, prospective and cross-sectional studies are not suitable for determining any causal impact. Methodological limitations were also noted in prospective and cross-sectional studies that were based on self-reported data, in particular dietary consumption, which are prone to subjectivity, measurement errors, and desirability biases.^17–26^ The two cross-sectional studies reported the small sample size as a limitation.^19^^,^^25^ In the 3 prospective studies, short follow-up duration was stated as a limitation,^17^^,^^18^^,^^20^ and a lack of information on the type of products consumed was reported in the 2 prospective studies.^20^^,^^21^ Limitations on health markers or outcomes were reported. According to Torjusen et al,^20^ an additional limitation was related to the lack of biological measures to estimate the level of pesticide residues. Sun et al^26^ did not distinguish between type 1 and type 2 diabetes and considered the data on OF purchase to be indicators of consumption. Finally, limitations were related to certain possible confounding factors that are not taken into account in studies. The prospective study based on the Norwegian Mother and Child Cohort Study^20^ included the following variables as potential confounders: maternal prepregnancy BMI, gestational weight gain, maternal age, education, income, smoking, dietary intake, and exercise. The authors concluded that the available data do not make it possible to assess the impact of prepregnancy nutritional status, which may be a confounder in the relationship shown. In prospective studies based on the NutriNet-Santé French cohort,^17^^,^^18^ multiple confounding factors (ie, sociodemographic, lifestyles and dietary patterns) were considered, but residual factors resulting from unmeasured factors or inaccuracy in the assessment of some covariates may have influenced the observed associations. In the French NutriNet-Santé studies,^17^^,^^18^^,^^24^ the authors agreed on the limitation related to the participants’ profile. It was reported that NutriNet Santé participants are more often young, female, had a higher level of formal education, and had healthier dietary patterns than the general population.^30^ In both interventional studies,^27^^,^^28^ limitations were related to the difference in FV varieties, the quantity of dietary intakes or dietary patterns compared between organic and conventional groups, the small sample size, and the short follow-up duration. Grinder-Pederson et al^27^ also reported a restricted number of biomarkers measured and protocols that differ, which makes the comparison of results complicated and inconclusive.

However, despite the numerous limitations, the studies included in this review also have strengths, mainly related to the sample (size, representativity of the population), protocol, and follow-up duration. Three studies reported the large sample size as a strength,^17^^,^^18^^,^^21^ and 4 highlighted the representativeness of the sample population (eg, participants with different lifestyles and eating habits, wide range of covariables considered).^22^^,^^23^^,^^25^^,^^26^ Other strengths were related to the detailed information about participant diet and the integration of the main potential confounding factors.^20–22^^,^^25^ Baudry et al^24^ explained that biological measurements (ie, total serum cholesterol, high-density lipoprotein cholesterol, low-density lipoprotein cholesterol, serum triglycerides, and fasting blood glucose) were accurate and consistent. A long duration of follow-up was reported by Bradbury et al^21^ (participants were followed up for 12 years), and the originality of the health outcome studied was highlighted in2 articles.^24^^,^^25^

The conclusion of the present review is consistent with other systematic reviews and meta-analyses. The systematic review of Dangour et al,^11^ comparable to this work and covering 2 studies from the present review,^22^^,^^27^ showed no difference in nutrition-related health outcomes between OF and CF exposures but suggested an association between consuming strictly organic dairy products and a reduced risk of eczema in infants. The second review, by Vigar et al,^13^ which included 6 studies we also include in the present work,^17^^,^^20–22^^,^^24^^,^^27^ concluded that although observational studies found that OF intake was associated with significant positive outcomes on MetS, BMI, non-Hodgkin’s lymphoma, preeclampsia, and infertility, the current evidence base does not permit a definitive statement on the long-term health benefits of intake. The authors highlighted that the consumption of OF is often tied to overall healthier dietary behaviors that are likely to influence the results. Hurtado-Barroso et al^28^ suggested a quite similar conclusion: evidence is still scarce concerning the impact of OF intake on health, but OF seems to contribute to maintaining an optimal health status and decreases the risk of chronic disease. Smith-Spangler et al^12^ identified only 3 human studies examining clinical outcomes, and none of those reported significant differences between populations by food type for allergic outcomes or symptomatic *Campylobacter* infection. The authors highlighted the heterogeneous and limited number of studies and the presence of publication bias. In their perspective study, which included 1 study from the present review^22^ and focused more specifically on children’s health, Batra et al^31^ reported that there was no evidence of a positive health effect when children ate OF compared with CF. In their review, Crinnion et al,^32^ covering 2 studies from the present review,^22^^,^^27^ concluded that although in vitro studies consistently demonstrate that organic FV have greater antioxidant activity, are more potent suppressors of the mutagenic action of toxic compounds, and inhibit the proliferation of certain cancer cell lines, in vivo studies of antioxidant activity in humans have not demonstrated additional benefit. However, clear health benefits from consuming organic dairy products have been demonstrated regarding allergic dermatitis. In contrast, Johansson et al^33^ concluded in their review that both animal and in vitro studies clearly indicate the benefits of consumption of OF instead of CF, yet investigations in humans are scarce, and only few of those performed can confirm positive public health benefits related to consuming OF. The authors highlighted that OF health benefits are unclear because specific large amounts of nutritionally high-value compounds with high antioxidant capacity do not seem to be the key for improved public health from OF consumption. Instead, synergistic effects of several constituents might underlie their possible positive effects.

Some of the uncertainties associated with studies in humans stem from the difficulty of accurately determining the consumption of organic FV compared with equivalent conventional products. In addition, it is rarely possible to establish a link between any observed effect and the farming practices responsible for that effect, such as soil amendment or crop treatment. These parameters are easier to control in animal experiments, where both organic and conventional products are of the same cultivars and originate from neighboring farms with comparable pedoclimatic conditions, enabling feeding tests based on factorial trials. As previously suggested by Lauridsen et al,^34^ Srednicka-Tober et al,^35^ and Barański et al,^36^ both fertilization management (mineral fertilizer–based protocols used in conventional farming vs composted manure inputs according to organic farming standards) and crop protection practices (pesticide-based protocols used in conventional agriculture vs crop protection according to organic standards) may affect the physiological status of mammals (Table S4). These authors attempted to explain the changes observed by the substantial differences in composition of nutrients, micronutrients, residues, or contaminants between foods produced by organic and conventional agriculture.^34^ Although several of these explanations are plausible, they nonetheless remain hypotheses that need to be explored.

Animal experiments also offer the possibility of monitoring many outcomes, as reported by Dangour et al^11^ and Velimirov et al^37^ Moreover, in many cases, animal studies provide an opportunity to explore the mechanisms behind physiological effects or dysfunctions and to suggest avenues for future research. Velimirov et al^37^ found that laboratory experiments tended to show a positive effect of organic feed compared with conventional feed, particularly on reproductive performance and immune responses. However, the authors questioned the relevance of the investigated biomarkers in terms of human health and emphasized the need to confirm these results both in laboratory animals and in humans. Analysis of the data published in recent years led to similar conclusions, confirming the impact of cultivation methods on various biomarkers but without establishing a confident causal relationship between OF and health.^35^^,^^36^^,^^38^ These studies demonstrated that in rodents and birds, immune system parameters may be modulated according to feed composition (Table S4). However, as reported by Van Norman et al,^39^ the responses of the animal immune system do not accurately predict those of the human immune system. Animal studies underlined the role of agricultural practices on foodstuff composition, and they converged on highlighting the sensitivity to different diets of certain biomarkers associated with the immune, hormonal, or metabolic systems. They also provided mechanistic interpretability of the observed effects, among which epigenetic modifications were suggested to explain transgenerational changes.^36^ Although several of these explanations are plausible, they nonetheless remain hypotheses that need to be explored. Biomarkers and outcomes should be interpreted carefully. Targeted experiments carried out in vivo or in vitro would be a major contribution in reducing uncertainties and improving a weight of evidence approach.

### Limitations

The studies included in this review did not focus specifically on FV but on diet in general, the low number of studies available that enabled us to assess the direct health benefits of OF, the possibility of not identifying all the relevant publications, and bias following the exclusion of articles published in a language other than English. Difficulties were encountered in extracting the intake of organic FV category and it was revealed that the quality of FV varies widely according to different factors. Sensory and nutritional qualities of FV are mainly influenced by the choice of variety and genotype,^10^ followed by the climate, farming conditions, and the stage of ripeness at the time of picking. Pedoclimatic conditions and production techniques, particularly for crops, can have a marginal effect on antioxidant composition, with slightly higher concentrations in organic produce.^36^ The determinism of individual health remains complex,^40^ and even though the direct and indirect health benefits of diet have been well documented, it remains difficult to conclude that 1 production method is more favorable than another.

### Strengths

The first strength of the present review is the use of the Jadad score and the French National Authority for Health method to evaluate the degree of study reliability and the level of evidence in the literature, respectively. Second, 7 reviewers with different and complementary expertise consulted with each other to agree on a consensus when analyzing the articles. Although most similar works are based on nutrient and pesticide residue contents in OF and/or CF, the present review distinguishes itself from others by focusing only on the direct health impacts of consuming OF or CF with broad inclusion criteria. Therefore, all types of studies indicating indirect health benefit were excluded, such as in vitro studies, as well as those only assessing values (eg, nutrient, pesticide residues). And third, 3 animal studies were included in our discussion to support the analysis of data from human studies, which is an original aspect of the present review.

## CONCLUSIONS

This systematic review did not demonstrate a greater health benefit of a diet based on products from organic farming compared with those from conventional farming. As in previous reviews, it was concluded that there is a paucity of available data and considerable heterogeneity in study designs (namely, participants, exposures, durations, health outcomes, and residual confounding factors). Well-designed interventional studies (ie, with direct measurement of contaminants and equal dietary intakes) are required. Long-term intervention studies are also needed. Due to possible epigenetic mechanisms, particular attention should be paid to the vulnerable window of life, such as the 1000 days period. The combination of traditional measurements of physiological or blood parameters and overall approaches based on systems biology, such as metabolomics, as previously proposed^38^ is probably an approach to be encouraged in studies. The literature clearly shows that products resulting from agroecology practices, including organic farming, could reduce direct exposure to harmful substances such as pesticide residues, particularly for vulnerable consumers (eg, women of childbearing age, pregnant women, children). However, the affordability of this type of food remains a challenge for these consumers. Further developing these production practices would make them affordable and accessible to these most vulnerable populations and, consequently, to the general population.

## Supplementary Material

nuae104_Supplementary_Data
