# Supplementary material for: Potential Health Benefits of a Diet Rich in Organic Fruit and Vegetables versus a Diet Based on Conventional Produce: A Systematic Review
Source: Nutr Rev. 2024 Aug 5;83(3):e1101–14. doi: 10.1093/nutrit/nuae104 (PMC11819487; doi:10.1093/nutrit/nuae104)
Supplement: nuae104_Supplementary_Data [file nuae104_supplementary_data.docx]

**Supplementary data:**

**Supplementary Table S1. PRISMA 2020 Checklist**

| **Section and Topic** | **Item #** | **Checklist item** | **Reported on page #** |
| --- | --- | --- | --- |
| **TITLE** | | |  |
| Title | 1 | Identify the report as a systematic review. | 1 |
| **ABSTRACT** | | |  |
| Abstract | 2 | See the PRISMA 2020 for Abstracts checklist. | 1-2 |
| **INTRODUCTION** | | |  |
| Rationale | 3 | Describe the rationale for the review in the context of existing knowledge. | 3-4 |
| Objectives | 4 | Provide an explicit statement of the objective(s) or question(s) the review addresses. | 3-4 |
| **METHODS** | | |  |
| Eligibility criteria | 5 | Specify the inclusion and exclusion criteria for the review and how studies were grouped for the syntheses. | 4-5 |
| Information sources | 6 | Specify all databases, registers, websites, organisations, reference lists and other sources searched or consulted to identify studies. Specify the date when each source was last searched or consulted. | 4 |
| Search strategy | 7 | Present the full search strategies for all databases, registers and websites, including any filters and limits used. | 4-5 |
| Selection process | 8 | Specify the methods used to decide whether a study met the inclusion criteria of the review, including how many reviewers screened each record and each report retrieved, whether they worked independently, and if applicable, details of automation tools used in the process. | 4-5 |
| Data collection process | 9 | Specify the methods used to collect data from reports, including how many reviewers collected data from each report, whether they worked independently, any processes for obtaining or confirming data from study investigators, and if applicable, details of automation tools used in the process. | 4-5 |
| Data items | 10a | List and define all outcomes for which data were sought. Specify whether all results that were compatible with each outcome domain in each study were sought (e.g. for all measures, time points, analyses), and if not, the methods used to decide which results to collect. | 4-5 |
|  | 10b | List and define all other variables for which data were sought (e.g. participant and intervention characteristics, funding sources). Describe any assumptions made about any missing or unclear information. | 4-5 |
| Study risk of bias assessment | 11 | Specify the methods used to assess risk of bias in the included studies, including details of the tool(s) used, how many reviewers assessed each study and whether they worked independently, and if applicable, details of automation tools used in the process. | 4 |
| Effect measures | 12 | Specify for each outcome the effect measure(s) (e.g. risk ratio, mean difference) used in the synthesis or presentation of results. | 7, 8 and Table 2 (pages 10-25) |
| Synthesis methods | 13a | Describe the processes used to decide which studies were eligible for each synthesis (e.g. tabulating the study intervention characteristics and comparing against the planned groups for each synthesis (item #5)). | 4-5 |
|  | 13b | Describe any methods required to prepare the data for presentation or synthesis, such as handling of missing summary statistics, or data conversions. | N/A |
|  | 13c | Describe any methods used to tabulate or visually display results of individual studies and syntheses. | Table 2 (pages 10-25) |
|  | 13d | Describe any methods used to synthesize results and provide a rationale for the choice(s). If meta-analysis was performed, describe the model(s), method(s) to identify the presence and extent of statistical heterogeneity, and software package(s) used. | N/A |
|  | 13e | Describe any methods used to explore possible causes of heterogeneity among study results (e.g. subgroup analysis, meta-regression). | N/A |
|  | 13f | Describe any sensitivity analyses conducted to assess robustness of the synthesized results. | N/A |
| Reporting bias assessment | 14 | Describe any methods used to assess risk of bias due to missing results in a synthesis (arising from reporting biases). | 5 |
| Certainty assessment | 15 | Describe any methods used to assess certainty (or confidence) in the body of evidence for an outcome. | 5 and Supp table S3. |
| **RESULTS** | | |  |
| Study selection | 16a | Describe the results of the search and selection process, from the number of records identified in the search to the number of studies included in the review, ideally using a flow diagram. | 6 (figure 1) |
|  | 16b | Cite studies that might appear to meet the inclusion criteria, but which were excluded, and explain why they were excluded. | 5-6 |
| Study characteristics | 17 | Cite each included study and present its characteristics. | Table 2 (pages 10-25) and Supp Table S4. |
| Risk of bias in studies | 18 | Present assessments of risk of bias for each included study. | Supp. Table 3 |
| Results of individual studies | 19 | For all outcomes, present, for each study: (a) summary statistics for each group (where appropriate) and (b) an effect estimate and its precision (e.g. confidence/credible interval), ideally using structured tables or plots. | Table 2 (pages 10-25) and Supp Table S4. |
| Results of syntheses | 20a | For each synthesis, briefly summarise the characteristics and risk of bias among contributing studies. | 8-9 ; 26-27 |
|  | 20b | Present results of all statistical syntheses conducted. If meta-analysis was done, present for each the summary estimate and its precision (e.g. confidence/credible interval) and measures of statistical heterogeneity. If comparing groups, describe the direction of the effect. | N/A |
|  | 20c | Present results of all investigations of possible causes of heterogeneity among study results. | 35-36 |
|  | 20d | Present results of all sensitivity analyses conducted to assess the robustness of the synthesized results. | N/A |
| Reporting biases | 21 | Present assessments of risk of bias due to missing results (arising from reporting biases) for each synthesis assessed. | 8-9 ; 26-27 |
| Certainty of evidence | 22 | Present assessments of certainty (or confidence) in the body of evidence for each outcome assessed. | 8-9 ; 26-27 |
| **DISCUSSION** | | |  |
| Discussion | 23a | Provide a general interpretation of the results in the context of other evidence. | 27-29 |
|  | 23b | Discuss any limitations of the evidence included in the review. | 26-27 |
|  | 23c | Discuss any limitations of the review processes used. | 30-31 |
|  | 23d | Discuss implications of the results for practice, policy, and future research. | 31 |
| **OTHER INFORMATION** | | |  |
| Registration and protocol | 24a | Provide registration information for the review, including register name and registration number, or state that the review was not registered. | 4 |
|  | 24b | Indicate where the review protocol can be accessed, or state that a protocol was not prepared. | 4-6 |
|  | 24c | Describe and explain any amendments to information provided at registration or in the protocol. | N/A |
| Support | 25 | Describe sources of financial or non-financial support for the review, and the role of the funders or sponsors in the review. | 31 |
| Competing interests | 26 | Declare any competing interests of review authors. | 31 |
| Availability of data, code and other materials | 27 | Report which of the following are publicly available and where they can be found: template data collection forms; data extracted from included studies; data used for all analyses; analytic code; any other materials used in the review. | 31, 35 |

**Supplementary Table S2.** Queries executed on Web of Science and PubMed

| TS = (ORGANIC NEAR/5 (FRUIT OR VEGETABLE) AND HUMAN HEALTH NOT (CONSUMER OR BEHAVIOUR OR PERCEPTION OR CELLS OR ORGANOLEPTIC))  TS = (ORGANIC NEAR/5 (FRUIT OR VEGETABLE) AND (DIET* OR NUTRI*) AND HUMAN HEALTH NOT (CONSUMER OR BEHAVIOUR OR PERCEPTION OR CELLS OR ORGANOLEPTIC))  TS = (ORGANIC NEAR/5 (APPLE* OR TOMATO* OR BANANA* OR ORANGE* OR CARROT* OR MELON* OR PEACH* OR SALAD* OR ZUCCHINI* OR PEAR* OR CUCUMBER* OR GRAPE* OR KIWI* OR STRAWBERRY* OR APRICOT*) AND HUMAN HEALTH NOT (CONSUMER OR BEHAVIOUR OR PERCEPTION OR CELLS OR ORGANOLEPTIC))  TS = (ORGANIC NEAR/5 (APPLE* OR TOMATO* OR BANANA* OR ORANGE* OR CARROT* OR MELON* OR PEACH* OR SALAD* OR ZUCCHINI* OR PEAR* OR CUCUMBER* OR GRAPE* OR KIWI* OR STRAWBERRY* OR APRICOT*) AND (DIET OR NUTRI) AND HUMAN HEALTH NOT (CONSUMER OR BEHAVIOUR OR PERCEPTION OR CELLS OR ORGANOLEPTIC))  TS = (ORGANIC NEAR/5 (FRUIT OR VEGETABLE) AND PESTICID* NOT (CONSUMER OR BEHAVIOUR OR PERCEPTION OR CELLS OR ORGANOLEPTIC))  TS = (ORGANIC NEAR/5 (FRUIT OR VEGETABLE) AND MYCOTOXIN* NOT (CONSUMER OR BEHAVIOUR OR PERCEPTION OR CELLS OR ORGANOLEPTIC))  TS = (ORGANIC NEAR/5 (FRUIT OR VEGETABLE) AND HEAVY METAL* NOT (CONSUMER OR BEHAVIOUR OR PERCEPTION OR CELLS OR ORGANOLEPTIC))  TS = (ORGANIC NEAR/5(FRUIT OR VEGETABLE) AND VITAMIN* NOT (CONSUMER OR BEHAVIOUR OR PERCEPTION OR CELLS OR ORGANOLEPTIC))  TS = (ORGANIC NEAR/5 (FRUIT OR VEGETABLE) AND MINERAL* NOT (CONSUMER OR BEHAVIOUR OR PERCEPTION OR CELLS OR ORGANOLEPTIC))  TS = (ORGANIC NEAR/5 (FRUIT OR VEGETABLE) AND FIBER* NOT (CONSUMER OR BEHAVIOUR OR PERCEPTION OR CELLS OR ORGANOLEPTIC))  TS = (ORGANIC NEAR/5 (FRUIT OR VEGETABLE) AND POLYPHENOL* NOT (CONSUMER OR BEHAVIOUR OR PERCEPTION OR CELLS OR ORGANOLEPTIC))  TS = (ORGANIC NEAR/5 (FRUIT OR VEGETABLE) AND DISEASE* NOT (CONSUMER OR BEHAVIOUR OR PERCEPTION OR CELLS OR ORGANOLEPTIC))  TS = (CONVENTIONAL NEAR/10 (FRUIT OR VEGETABLE) AND HUMAN HEALTH NOT (CONSUMER OR BEHAVIOUR OR PERCEPTION OR CELLS OR ORGANOLEPTIC))  TS = (CONVENTIONAL NEAR/10 (FRUIT OR VEGETABLE) AND (DIET* OR NUTRI*) AND HUMAN HEALTH NOT (CONSUMER OR BEHAVIOUR OR PERCEPTION OR CELLS OR ORGANOLEPTIC))  TS = (CONVENTIONAL NEAR/10 (APPLE* OR TOMATO* OR BANANA* OR ORANGE* OR CARROT* OR MELON* OR PEACH* OR SALAD* OR ZUCCHINI* OR PEAR* OR CUCUMBER* OR GRAPE* OR KIWI* OR STRAWBERRY* OR APRICOT*) AND HUMAN HEALTH NOT (CONSUMER OR BEHAVIOUR OR PERCEPTION OR CELLS OR ORGANOLEPTIC))  TS = (CONVENTIONAL NEAR/10 (APPLE* OR TOMATO* OR BANANA* OR ORANGE* OR CARROT* OR MELON* OR PEACH* OR SALAD* OR ZUCCHINI* OR PEAR* OR CUCUMBER* OR GRAPE* OR KIWI* OR STRAWBERRY* OR APRICOT*) AND (DIET OR NUTRI) AND HUMAN HEALTH NOT (CONSUMER OR BEHAVIOUR OR PERCEPTION OR CELLS OR ORGANOLEPTIC))  TS = (CONVENTIONAL NEAR/10 (FRUIT OR VEGETABLE) AND PESTICID* NOT (CONSUMER OR BEHAVIOUR OR PERCEPTION OR CELLS OR ORGANOLEPTIC))  TS = (CONVENTIONAL NEAR/10 (FRUIT OR VEGETABLE) AND MYCOTOXIN* NOT (CONSUMER OR BEHAVIOUR OR PERCEPTION OR CELLS OR ORGANOLEPTIC))  TS = (CONVENTIONAL NEAR/10 (FRUIT OR VEGETABLE) AND HEAVY METAL* NOT (CONSUMER OR BEHAVIOUR OR PERCEPTION OR CELLS OR ORGANOLEPTIC))  TS = (CONVENTIONAL NEAR/10 (FRUIT OR VEGETABLE) AND VITAMIN* NOT (CONSUMER OR BEHAVIOUR OR PERCEPTION OR CELLS OR ORGANOLEPTIC))  TS = (CONVENTIONAL NEAR/10 (FRUIT OR VEGETABLE) AND MINERAL* NOT (CONSUMER OR BEHAVIOUR OR PERCEPTION OR CELLS OR ORGANOLEPTIC))  TS = (CONVENTIONAL NEAR/10 (FRUIT OR VEGETABLE) AND FIBER* NOT (CONSUMER OR BEHAVIOUR OR PERCEPTION OR CELLS OR ORGANOLEPTIC))  TS = (CONVENTIONAL NEAR/10 (FRUIT OR VEGETABLE) AND POLYPHENOL* NOT (CONSUMER OR BEHAVIOUR OR PERCEPTION OR CELLS OR ORGANOLEPTIC))  TS = (CONVENTIONAL NEAR/10 (FRUIT OR VEGETABLE) AND DISEASE* NOT (CONSUMER OR BEHAVIOUR OR PERCEPTION OR CELLS OR ORGANOLEPTIC))  TS = ((CONVENTIONAL OR ORGANIC) NEAR/2 (FRUIT OR VEGETABLE) NOT (CONSUMER OR BEHAVIOUR OR PERCEPTION OR CELLS OR ORGANOLEPTIC))  TS = ((CONVENTIONAL OR ORGANIC) NEAR/2 (FRUIT OR VEGETABLE) AND (HUMAN NEAR/1 HEALTH) NOT (CONSUMER OR BEHAVIOUR OR PERCEPTION OR CELLS OR ORGANOLEPTIC))  TS = ((CONVENTIONAL OR ORGANIC) NEAR/2 (FRUIT OR VEGETABLE) AND (DIET* OR NUTRI*) AND HUMAN HEALTH NOT (CONSUMER OR BEHAVIOUR OR PERCEPTION OR CELLS OR ORGANOLEPTIC))  TS = ((CONVENTIONAL OR ORGANIC) NEAR/2 (APPLE* OR TOMATO* OR BANANA* OR ORANGE* OR CARROT* OR MELON* OR PEACH* OR SALAD* OR ZUCCHINI* OR PEAR* OR CUCUMBER* OR GRAPE* OR KIWI* OR STRAWBERRY* OR APRICOT*) AND HUMAN HEALTH NOT (CONSUMER OR BEHAVIOUR OR PERCEPTION OR CELLS OR ORGANOLEPTIC))  TS = ((CONVENTIONAL OR ORGANIC) NEAR/10 (APPLE* OR TOMATO* OR BANANA* OR ORANGE* OR CARROT* OR MELON* OR PEACH* OR SALAD* OR ZUCCHINI* OR PEAR* OR CUCUMBER* OR GRAPE* OR KIWI* OR STRAWBERRY* OR APRICOT*) AND (DIET* OR NUTRI*) AND HUMAN HEALTH NOT (CONSUMER OR BEHAVIOUR OR PERCEPTION OR CELLS OR ORGANOLEPTIC)) |
| --- |

**Supplementary Table S3.** JADAD score and HAS grade for the eligible studies in humans.

|  |  | **Baudry, 2018** ^17^ | **Kesse-Guyot, 2020** ^18^ | **Simões-Wüst, 2017** ^19^ | **Bradbury, 2014** ^21^ | **Torjusen, 2014** ^20^ | **Kummel-ing, 2008** ^22^ | **Ludwig-Borycz, 2021** ^23^ | **Sun, 2018** ^26^ | **Baudry, 2018** ^24^ | **Gosling, 2021** ^25^ | **Grinder-Pedersen, 2003** ^27^ | **Hurtado-Barroso, 2019** ^28(p)^ |
| --- | --- | --- | --- | --- | --- | --- | --- | --- | --- | --- | --- | --- | --- |
| **JADAD Scale (scale from 0 to 5)** | | | | | | | | | | | | | |
| **Was the study described as randomised (this includes words such as randomly, random, and randomisation)?** | Yes:1 No: 0 | 1 | 0 | 0 | 0 | 0 | 0 | 0 | 0 | 0 | 0 | 1 | 1 |
| **Was the method used to generate the sequence of randomisation described and appropriate (table of random numbers, computer-generated, etc)?** | Yes:1 No: 0 | 1 | 0 | 0 | 0 | 0 | 0 | 0 | 0 | 0 | 0 | 1 | 1 |
| **Was the study described as double blind?** | Yes:1 No: 0 | 0 | 0 | 0 | 0 | 0 | 0 | 0 | 0 | 0 | 0 | 1 | 0 |
| **Was the method of double blinding described and appropriate (identical placebo, active placebo, dummy, etc)?** | Yes:1 No: 0 | 0 | 0 | 0 | 0 | 0 | 0 | 0 | 0 | 0 | 0 | 1 | 0 |
| **Was there a description of withdrawals and dropouts?** | Yes:1 No: 0 | 1 | 0 | 0 | 0 | 0 | 1 | 0 | 0 | 0 | 0 | 0 | 1 |
| **Deduct one point if the method used to generate the sequence of randomisation was described and it was inappropriate (patients were allocated alternately, or according to date of birth, hospital number, etc).** | Yes: -1 No: 0 | 0 | 0 | 0 | 0 | 0 | 0 | 0 | 0 | 0 | 0 | 0 | 0 |
| **Deduct one point if the study was described as double blind but the method of blinding was inappropriate (e.g., comparison of tablet vs. injection with no double dummy).** | Yes: -1 No: 0 | 0 | 0 | 0 | 0 | 0 | 0 | 0 | 0 | 0 | 0 | 0 | 0 |
| **TOTAL JADAD SCORE** | | **3** | **0** | **0** | **0** | **0** | **1** | **0** | **0** | **0** | **0** | **4** | **3** |
| **HAS Grade*** | | **C** | **C** | **C** | **C** | **C** | **C** | **C** | **C** | **B** | **C** | **B** | **B** |

* Higher Safety Authority (HAS) grades are from A to C, according to the following guidelines: Grade A=Scientific evidence is established (e.g., strong randomised, controlled, meta-analysis of randomised controlled trials, decision analysis based on well-conducted studies); Grade B=Scientific presumption provided by studies of intermediate level of evidence (e.g., weak randomised controlled trials, well-conducted non-randomised controlled studies, cohort studies, etc.); Grade C: Low level of scientific evidence (e.g., case-control studies, comparative studies with significant bias, retrospective studies, case series, descriptive epidemiological studies such as cross-sectional and longitudinal).

**Supplementary Table S4.** Animal studies (n = 3) analysed to support human studies.

| **Author, year** | **Study purpose** | **Model**  **characteristics** | **Size** | **Study design** | **Duration** | **Results** | **Conclusions** |
| --- | --- | --- | --- | --- | --- | --- | --- |
| **Srednicka-Tober, 2013** ^35^ | To test the effects of diets produced with feedstuffs whose constituents are derived from agricultural production using mineral fertilisers vs. organic fertilisers, with or without the use of plant protection products on: (a) the composition of the rats' diet, and (b) the body composition, growth and certain biochemical parameters of the rats. | Wistar rats  Experimental feed was provided *ad libitum* during mating, pregnancy, lactation of the parental rats and to young rats postweaning for a 9-week period. The effects of the diets were tested on young male rats. | 48 animals at the beginning of the experiment (32 females and 16 males) | Four diets were tested:  (a) Organic (organic manure x no treatments prohibited in organic)  (b) Mineral fertiliser x no treatments prohibited in organic farming  (c) Organic manure x conventional plant protection treatments  (d) Conventional (mineral fertiliser x conventional plant protection treatments)  At weaning, 6 male animals (randomly selected from 2 litters of females on the same diet) were placed in individual cages and fed the same diet as their parents for a further 9 weeks. Each diet was tested in 4 replicates. | Total exposure period = 23 weeks, as follows: 3 weeks before breeding + 8 weeks pregnancy + 3 weeks lactation + 9 weeks postweaning | The most significant effects are related to the fertilisation method and not to the crop protection method. Of all the parameters measured, those related to the immune system showed the most change.  The use of manure compared to mineral fertilisers is responsible for the most marked effects: a higher level of leukocytes (16%), plasma glucose (33%), leptin (29%), IGF1 (46%), corticosterone (18%) and spontaneous lymphocyte proliferation + a 121% increase in spontaneous lymphocyte proliferation, a 35% decrease in concanavalin-induced lymphocyte proliferation and a 4-fold decrease in lipopolysaccharide-induced lymphocyte proliferation. | Crop production practices (fertilisation and crop protection)  that modulate the composition of feeds, resulting in significant effects on animal physiology, especially immune status. |
| **Barański, 2021** ^36^ | To identify the effects of feeds made from organic and conventional crops on growth, hormonal and immune system parameters | Factorial two-generation dietary intervention trial carried out in Wistar rats.  Experimental feed was provided *ad libitum* to the animals. The effects of the diets were tested on young male rats sampled among F1 and F2. | 48 (32 females and 16 males) | A 2x2 factorial design was used to separate the effects of contrasting crop protection methods (use or non-use of synthetic chemical pesticides) and fertilisers (mineral nitrogen, phosphorus and potassium (NPK) fertilisers vs. manure use) applied in conventional and organic crop production. | Total duration = 23 weeks (F1) and 35 weeks (F2) | Conventional, pesticide-based crop protection resulted in significantly lower fibre, polyphenol, flavonoid, and lutein, but higher lipid, aldicarb and diquat concentrations in animal feeds.  Conventional, mineral NPK-based fertilisation resulted in significantly lower polyphenol, but higher cadmium and protein concentrations in feeds.  Feed composition differences had a significant effect on feed intake, weight gain, plasma hormone, immunoglobulin concentrations, and lymphocyte proliferation in F1 and F1 and on body weight at weaning in F2. | Relatively small changes in dietary intakes resulting from pesticide and/or mineral NPK-fertiliser use had complex and often interactive effects on endocrine, immune systems and growth parameters in rats. |
| **Huber, 2010** ^38^ | To determine the effects of production method on physiological and immune parameters of the chicken and identify health biomarkers to enable future studies in humans. | Chicken model (two-generation study) | 150 | A feeding experiment was performed in two generations of three groups of chickens differing in immune responsiveness, which were fed identically composed feeds from either organic or conventional produce. F2 was exposed to an immune challenge and euthanised at 13 weeks of age. | 13 weeks for animals of F2 | Significant differences were found between the groups: the conventionally fed chickens showed overall greater lifetime weight gain than the organically fed group, although feed intake was comparable. The animals organically fed showed better immune reactivity, a stronger response to immune challenge and slightly higher resistance to disease. | Diets from different origins, i.e., organic vs. conventional production systems, can induce physiological changes in two generations of chickens. |

F1, First generation; F2, Second generation
